# Surgical Management of Giant Basal Cell Carcinoma of the Upper Eyelid: A Case Report

**DOI:** 10.7759/cureus.103659

**Published:** 2026-02-15

**Authors:** Swatishree Nayak, Sumanjit Boro, Bifica Lyndogh

**Affiliations:** 1 Ophthalmology, All India Institute of Medical Sciences, Guwahati, IND; 2 Plastic Surgery, All India Institute of Medical Sciences, Guwahati, IND; 3 Pathology, All India Institute of Medical Sciences, Guwahati, IND

**Keywords:** basal cell carcinoma, eyelid reconstruction, infiltrative, surgical management, ulcerative

## Abstract

Basal cell carcinoma is the most common malignant tumor of the eyelid and, although typically slow growing, can become locally aggressive and disfiguring when diagnosis or treatment is delayed. We report a rare case of a giant, long-standing basal cell carcinoma of the upper eyelid in a 62-year-old female who presented with a 30-year history of a progressively enlarging pigmented ulcerative lesion associated with mechanical ptosis. Clinical examination revealed a large indurated ulcer with rolled margins involving the upper eyelid and eyebrow region, with no regional lymphadenopathy. Excisional biopsy followed by histopathological evaluation confirmed an infiltrative variant of basal cell carcinoma. The patient underwent wide local excision with adequate safety margins and immediate reconstruction using a forehead axial flap, with the donor site covered by a split-thickness skin graft. Postoperative recovery was uneventful, with good graft uptake and satisfactory functional and cosmetic outcomes at follow-up. This case highlights the potential for basal cell carcinoma to attain giant proportions when neglected, underscores the importance of histopathological confirmation and margin-controlled excision, and demonstrates that even extensive periocular tumors can be successfully managed with appropriate surgical planning and multidisciplinary reconstruction.

## Introduction

Basal cell carcinoma (BCC) is a common type of skin cancer in humans, often arising on sun-damaged skin [1]. Although rarely fatal, BCC can be destructive and disfiguring if left untreated or inadequately treated. Typically, BCC appears as flesh- or pink-colored, pearly papules with overlying ulcers or visible blood vessels. These lesions commonly develop on the head and neck but can also occur on the trunk and extremities. Unlike many cancers, BCC rarely metastasizes, meaning it does not spread to other parts of the body [2]. Herein, we present a case of BCC, which was a pigmented ulcerative lesion of long duration on the left upper eyelid in a sexagenarian female. The unusual location of the cancer and the effective management of this long-standing lesion warrant reporting. Although it rarely metastasizes, BCC can be locally aggressive and cause significant functional and cosmetic morbidity if left untreated. The periocular region is a common site, with eyelid involvement reported in 5-10% of cases [2].

BCC is the most frequent eyelid malignancy in Western populations; however, studies from India and other Asian countries show variable patterns, with sebaceous gland carcinoma and squamous cell carcinoma also contributing substantially [2]. This variability highlights the importance of reporting individual cases to better define regional disease burden and clinical behavior.

Clinically, BCC most commonly presents as a nodular lesion with pearly margins, but pigmented, ulcerative, and infiltrative variants are well recognized. Long-standing and infiltrative lesions are associated with greater local tissue destruction and pose significant reconstructive challenges. While the lower eyelid is most commonly involved, upper eyelid BCC is less frequent and often diagnosed late [2]. We report a rare case of a long-standing, giant, pigmented ulcerative basal cell carcinoma of the upper eyelid, managed successfully with surgical excision and reconstruction, to highlight the clinical spectrum and management challenges of advanced periocular BCC.

## Case presentation

**Figure 1:**
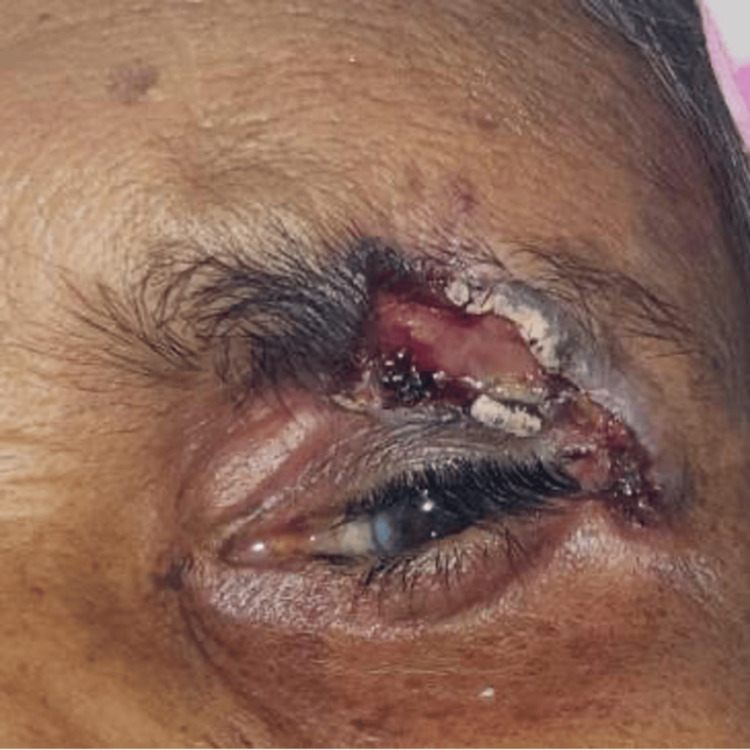
Pigmented ulcerated lesion of size 6x3 cm on left upper lid crease margin extending till left mid eyebrow with rolled out irregular crusted margins, pigmented edges and indurated floor.

A 62-year-old female patient, a farmer by occupation, presented with a 30-year history of a gradually progressive, pigmented ulcerative lesion on her left upper eyelid. The lesion had ruptured 15 years ago and was associated with mild pain. She had not undergone any treatment for the same. She had a history of frequent sunlight exposure but no history of radiation or chemical exposure, sudden weight loss, trauma, or other facial or neck lesions. Her medical history included hypertension, which was controlled with the tablet amlodipine 0.5 mg once daily, and she had undergone bilateral cataract surgery. On examination, the patient appeared healthy with a low socioeconomic status. Her visual acuity in the left eye was 6/12. Over the left upper eyelid, there was a pigmented ulcerative lesion measuring 6x3 cm, extending vertically from the left upper eyelid crease margin till the left mid eyebrow, and horizontally from the middle 1/3rd of the eyebrow till approximately 8 mm lateral and below the lateral canthus. This size is considerably larger than the typical periocular basal cell carcinoma (BCC), which commonly presents as lesions <2 cm, thereby qualifying it as a giant basal cell carcinoma. The ulcer had an indurated base and rolled out crusted margins (Figure 1).

The lesion was immobile and appeared to involve the underlying bone. Mechanical ptosis was present, with no lagophthalmos or ectropion. The lesion did not bleed on touch, had mild discharge, and the surrounding area appeared normal. There were no palpable lymph nodes in the neck. Anterior segment and fundus examinations were unremarkable. Although the lesion appeared clinically fixed, there were no signs suggestive of orbital invasion, such as proptosis, restriction of ocular movements, or sensory deficits. Preoperative imaging was not performed, as there were no clinical features indicating orbital or deep tissue involvement.

**Figure 2:**
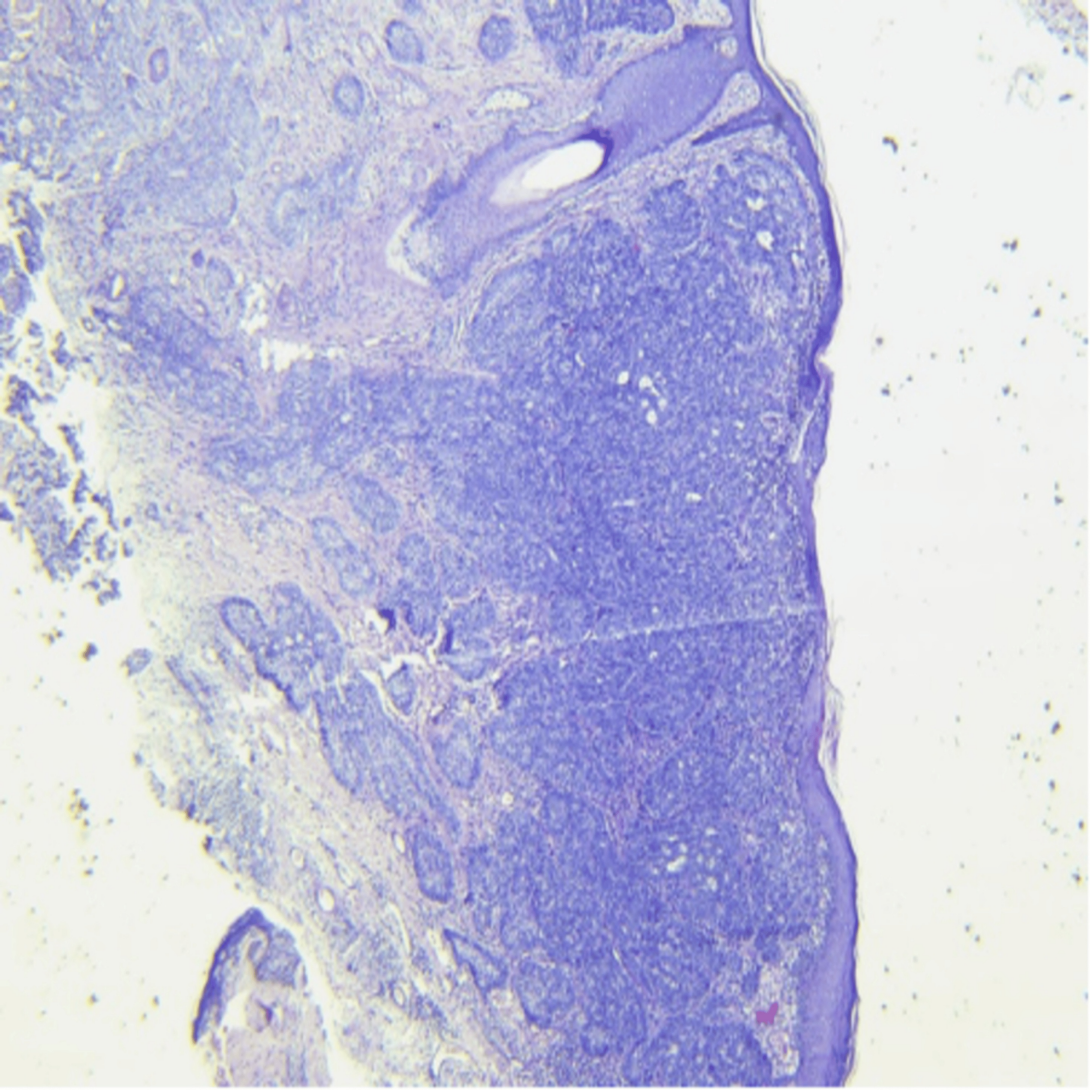
Histopathological examination revealed polygonal basaloid cells with hyperchromatic nuclei with peripheral palisading. Tumor cells were infiltrating deep into the dermis and reaching up to deep surgical margins consistent with a diagnosis of infiltrative BCC. BCC: basal cell carcinoma

**Figure 3:**
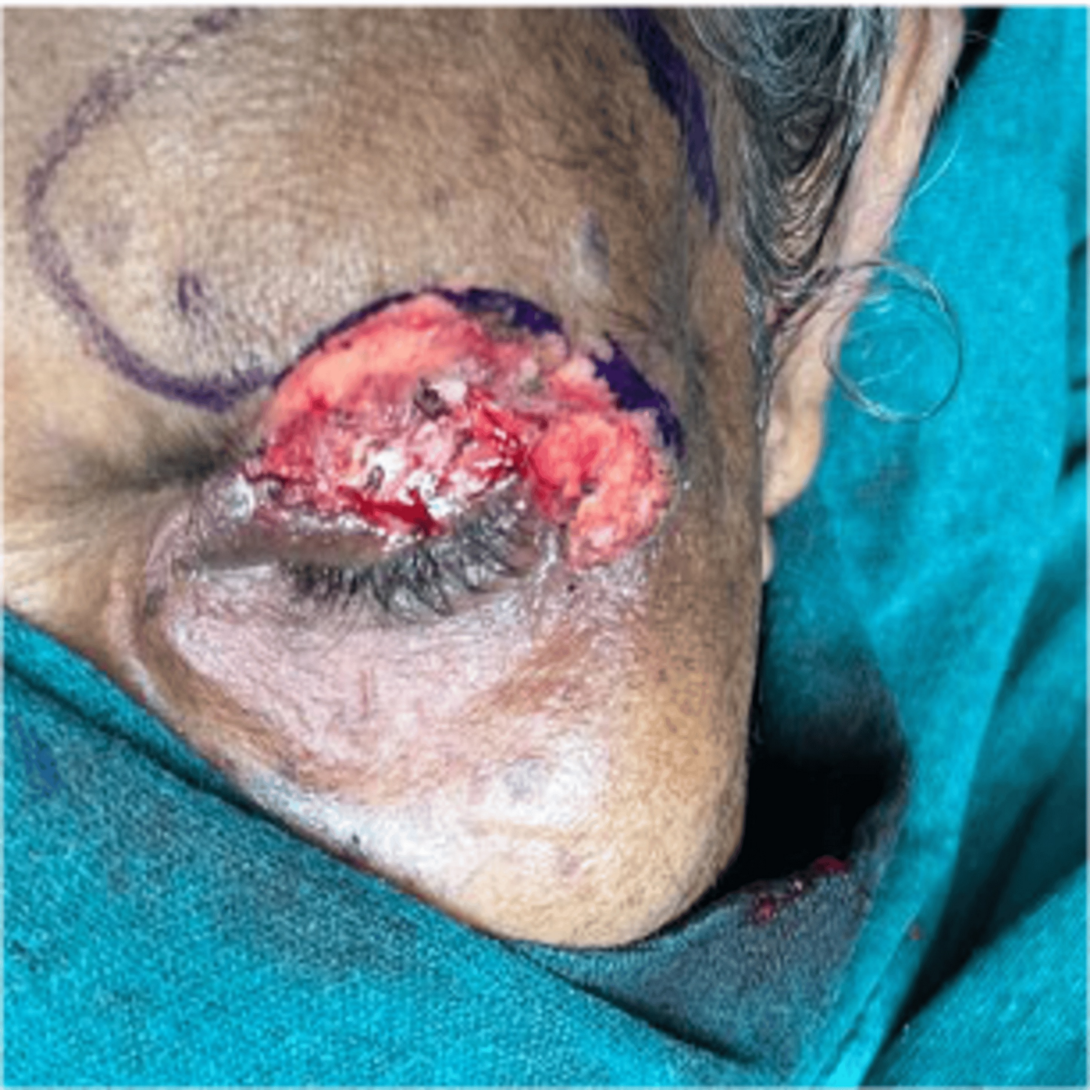
Intraoperative photos showing skin marking done around the ulcer maintaining 4 mm healthy margins. Incision given along the marking and ulcerative tissue along with 4 mm healthy skin excised.

The patient was planned for tumor excision biopsy and eyelid reconstruction under general anesthesia, along with the burns and plastic surgery team. Histopathological examination revealed polygonal basaloid cells with hyperchromatic nuclei and peripheral palisading. Tumor cells were infiltrating deep into the dermis and reaching deep surgical margins, consistent with a diagnosis of infiltrative BCC (Figure 2). Skin markings were placed, maintaining a 4 mm margin of clinically healthy tissue, consistent with standard recommendations for periocular BCC excision. The ulcerative lesion was excised en bloc (Figure 3).

**Figure 4:**
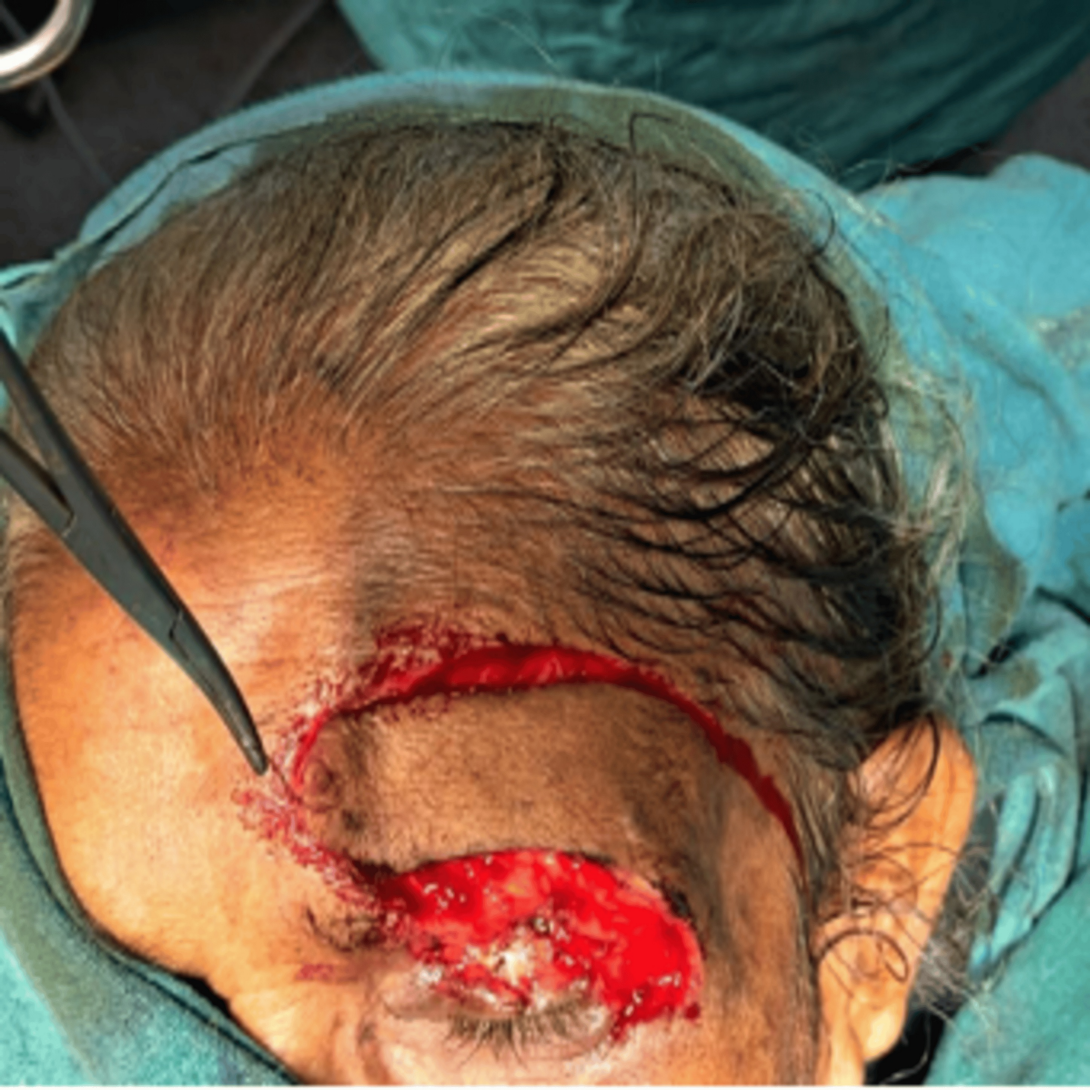
One axial pattern flap was harvested from forehead and transposed to the wound defect.

**Figure 5:**
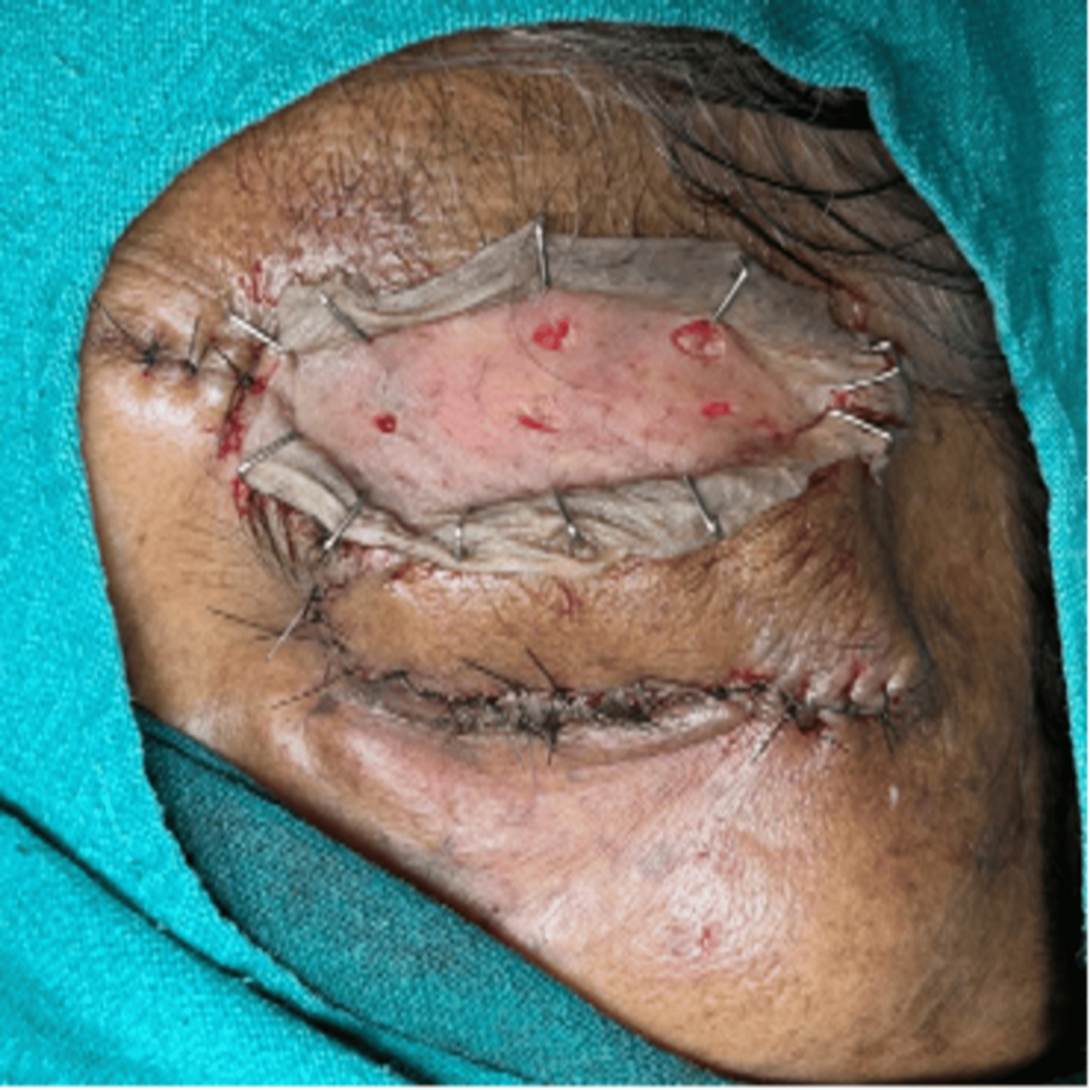
Raw area in the forehead was covered with skin graft which was harvested from the left thigh.

One axial pattern flap was harvested from the forehead and transposed to the wound defect (Figure 4). The raw area in the forehead was covered with a skin graft harvested from the left thigh (Figure 5). All surgical margins were free of tumor on final histopathological assessment. There was no evidence of perineural or lymphovascular invasion, and tumor infiltration was confined to the dermis without invasion of underlying muscle or bone.

**Figure 6:**
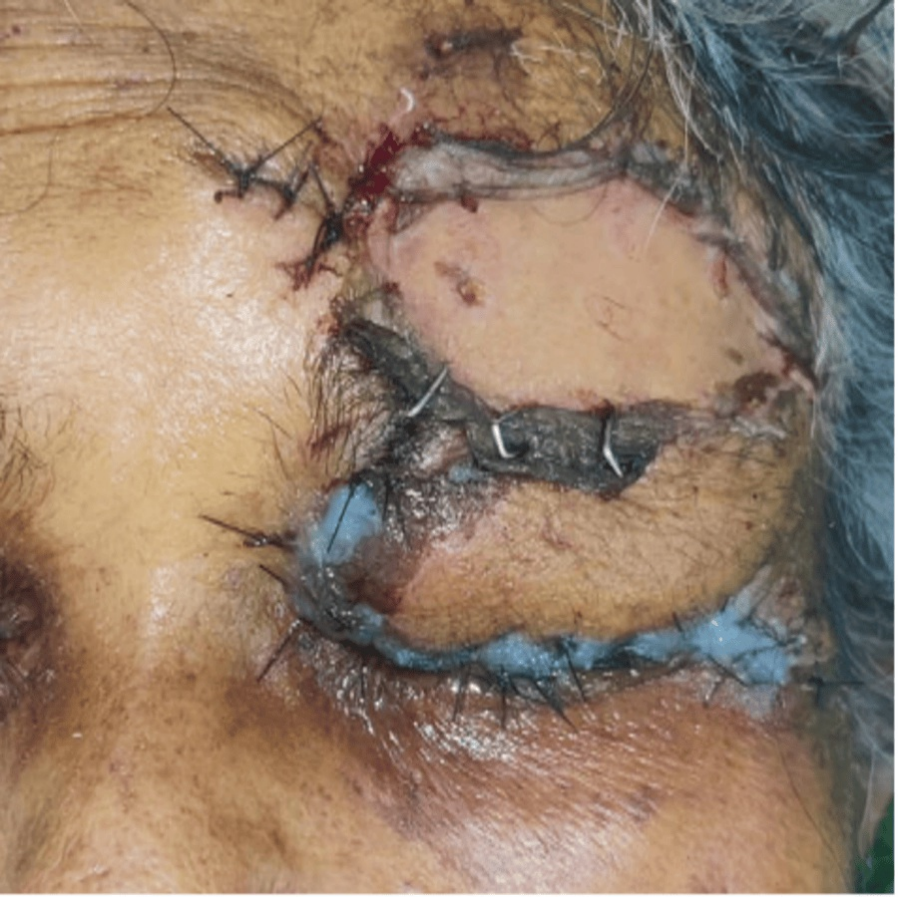
Postoperative day one photo: flap and graft were healthy, in position, sutures and pins were intact, lid edema was present with mild bleeding.

**Figure 7:**
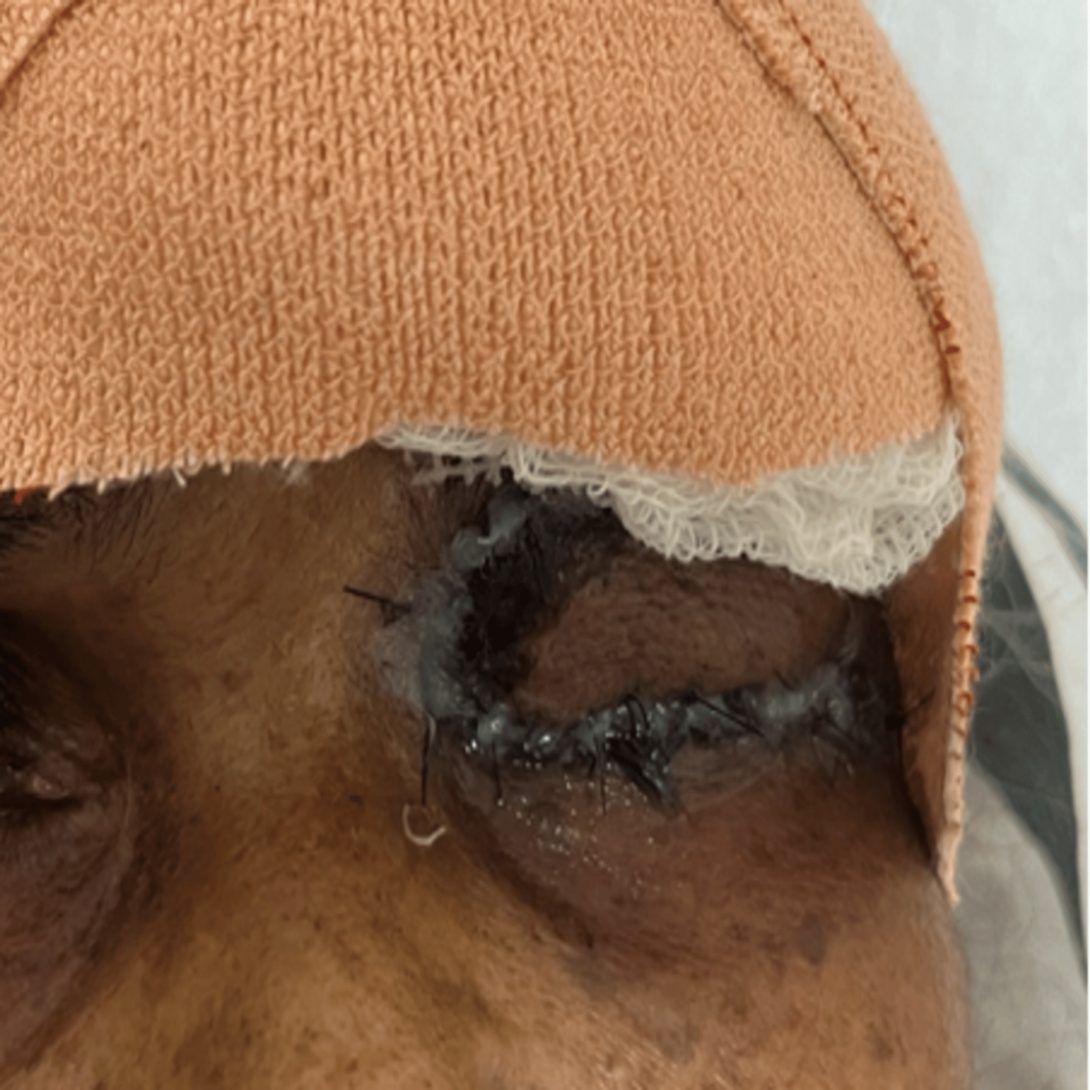
Postoperative day 12 photo showing mild lid edema, wound well apposed, healthy, sutures intact.

Postoperatively, the patient was started on inj. linezolid 600 mg IV OD, inj. Augmentin 1.2 g IV TDS and eye ointment moxifloxacin for local application over the wound. On postoperative day one, the flap and graft were healthy, and the sutures and pins were intact, with lid edema and mild bleeding (Figure 6). On postoperative day 12, the wound was well apposed with intact sutures (Figure 7).

**Figure 8:**
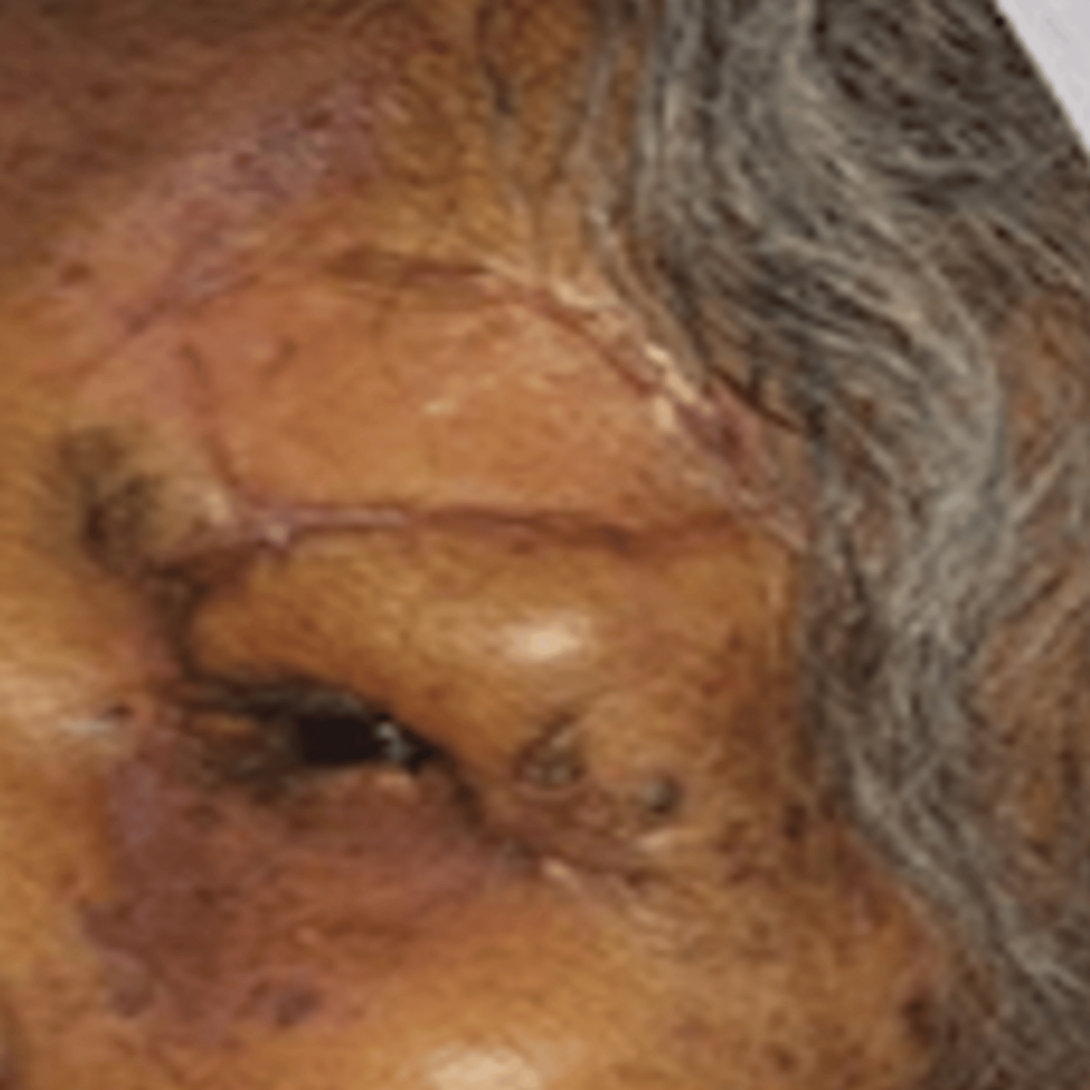
Postoperative day 30 photo showing graft well taken up and wound healing well.

On postoperative day 30, the graft was well-accepted, and the wound was healing well (Figure 8). The patient demonstrated adequate eyelid closure, stable visual acuity, a healthy ocular surface, and acceptable cosmetic outcome, with no evidence of early recurrence, and remains under regular follow-up.

## Discussion

Basal cell carcinoma (BCC) is one of the most common eyelid malignancies worldwide. Studies from Japan and China report a comparable incidence of squamous cell carcinoma (SCC) and BCC, while the pattern in India differs from Western populations and shows considerable variability [3,4]. Indian studies report conflicting distributions of eyelid malignancies as follows: Sihota et al. found BCC, SCC, and sebaceous gland carcinoma (SGC) to be nearly equally prevalent, whereas Kaliki et al. reported SGC as the predominant tumor [5,6]. A female predilection has been described, with a male-to-female ratio of approximately 1:1.5. Although the lower eyelid is most commonly affected, up to 25% of cases involve the upper eyelid [7].

BCC demonstrates diverse clinical and histological patterns with distinct biological behavior. While nodular BCC is the most common variant, infiltrative BCC is characterized by deeper dermal invasion and indistinct margins, contributing to increased local aggressiveness and recurrence risk [8,9]. Histologically, BCC may be undifferentiated (including pigmented, superficial, sclerosing, and infiltrative types) or differentiated toward adnexal structures [10,11]. Biopsy is essential for accurate diagnosis and subtype determination; our patient had infiltrative, undifferentiated BCC.

Management of BCC depends on tumor characteristics and patient factors. Available modalities include Mohs micrographic surgery (MMS), standard excision, electrodesiccation and curettage, radiotherapy, cryotherapy, topical agents, and systemic Hedgehog pathway inhibitors [12-14]. MMS remains the gold standard for high-risk and recurrent lesions due to complete margin control and tissue preservation [15,16]. For well-circumscribed tumors smaller than 2 cm, standard excision with approximately 4 mm margins is generally adequate. Radiotherapy and cryotherapy are reserved for selected cases but are limited by cosmetic outcomes and lack of histologic margin assessment [17]. Topical therapies are effective for superficial BCC but do not confirm complete tumor clearance, while systemic agents are reserved for advanced or metastatic disease [18-20].

The present case is notable for its giant size (6×3 cm), long duration, and upper eyelid involvement - features that are uncommon and associated with delayed diagnosis. Infiltrative BCCs are known to exhibit subclinical extension beyond visible margins, contributing to an increased risk of recurrence compared to nodular variants.

Surgical excision remains the mainstay of treatment for periocular BCC. For well-circumscribed lesions smaller than 2 cm, excision margins of 3-4 mm are generally considered adequate. However, infiltrative subtypes often warrant wider margins or margin-controlled excision. In the present case, a 4 mm margin was selected to balance oncologic clearance with preservation of critical periocular structures. Tumor-free margins were confirmed histologically.

Mohs micrographic surgery offers the highest cure rates for high-risk BCCs; however, its availability may be limited. Alternative modalities, such as radiotherapy, cryotherapy, and topical agents, have specific indications but do not allow histologic margin confirmation. Given the lesion size, infiltrative histology, and reconstructive requirements, wide local excision with flap reconstruction was considered the most appropriate approach in this patient. Although early postoperative outcomes were favorable, long-term follow-up is essential given the infiltrative histological subtype and the increased risk of local recurrence.

## Conclusions

This case illustrates that even long-standing, giant infiltrative basal cell carcinomas of the upper eyelid can be successfully managed with meticulous surgical excision and appropriate reconstruction. Accurate histopathological assessment, explicit margin clearance, and structured postoperative surveillance are critical to achieving optimal functional and cosmetic outcomes while minimizing recurrence risk.
